# Indications for carotid Doppler ultrasound in asymptomatic patients - are we ordering it correctly?

**DOI:** 10.1590/1677-5449.202200841

**Published:** 2023-08-07

**Authors:** Alcides Alves da Silva, André Brusamolin Moro, Jeferson Freitas Toregeani

**Affiliations:** 1 Centro Universitário da Fundação Assis Gurgacz - FAG, Cascavel, PR, Brasil.; 2 Universidade Estadual do Oeste do Paraná - UNIOESTE, Cascavel, PR, Brasil.

**Keywords:** asymptomatic carotid atherosclerosis, screening for asymptomatic carotid stenosis, carotid artery ultrasonography

## Abstract

**Background:**

Carotid arteries are frequently the site of pathologies, the most common being atherosclerosis, which may result in the formation of plaques, causing stenosis. Doppler ultrasound is currently the exam of choice for assessment of the carotid arteries in asymptomatic patients to screen for and diagnose vascular lesions. Current guidelines recommend screening patients who have risk factors for carotid stenosis and who are able and willing to undergo medical treatment and/or carotid intervention. Screening asymptomatic patients in the general adult population who have no significant risk factors is not recommended.

**Objectives:**

To assess whether medical experts rely on the literature to request Doppler ultrasound for screening.

**Methods:**

A retrospective selection of patients was performed based on requests for carotid ultrasound. The data collected were computed and analyzed using RStudio version 1.3.959.

**Results:**

The request was evaluated as appropriate as long as the patients presented at least one risk factor for carotid plaques. Fifty-five out of 152 patients met criteria for carotid screening. The most frequent indication in the study population was vascular check-up. Arterial hypertension was the most prevalent risk factor. Vascular surgery specialists were more likely to order the exam correctly (odds ratio for correct indications: 3.52 [CI 1.14 - 10.87], with p=0.02). The rate of correct Doppler ultrasound requests was 36% (95%CI 29 to 42%).

**Conclusions:**

An excess of requests for carotid ultrasound screening was found in this study. Vascular surgeons more often requested the test correctly.

## INTRODUCTION

The carotid arteries are frequently the site of pathological changes from birth to old age, the most common of which is stenosis caused by atherosclerosis.^1^ This pathology has a predilection for certain arteries, affecting the carotids, primarily at the level of the bifurcation and the initial portion of the internal carotid.^2^ Atherosclerosis can lead to formation of plaques with varied morphology and characteristics, which can cause stenosis and obstruct the carotid or result in embolization to the brain.^3-5^

The main risk factors for extracranial carotid disease include advanced age, systemic arterial hypertension (SAH), smoking, hypercholesterolemia, diabetes mellitus (DM), male sex, obesity, peripheral arterial occlusive disease (PAOD), coronary artery diseases (CAD), and cardiac diseases.^6-8^ Atherogenesis, followed by formation of atheromatous plaques, is related to cerebrovascular events.^9^

As a consequence of the degree of stenosis and embolization from the atheromatous plaque, cerebral blood flow may be compromised, limiting the supply of oxygen and glucose and possibly leading to stroke, transitory ischemic accident (TIA), amaurosis fugax, and other neurological conditions.^6^ Screening for carotid stenosis in asymptomatic patients is based on prevention of stroke, since it may be the first evidence of significant carotid atherosclerosis.^10^

Screening methods are essential in clinical practice because of the severity of long term undiagnosed carotid stenosis. According to the European Society for Vascular Surgery (ESVS) guideline, screening for asymptomatic carotid stenosis is indicated in patients with risk factors for atherosclerosis, in order to optimize control of these factors, reduce morbidity and mortality, and avoid invasive procedures.^11^ In 2011, the ESVS recommended that screening should be conducted for groups of patients that have risk factors for carotid artery stenosis, but these patients should be willing and able to undergo medical treatment and/or carotid intervention if significant stenosis is diagnosed.^12^

Doppler ultrasonography (US) is currently the method of choice for assessment of the carotids in asymptomatic patients. It is widely available and is recommended for screening and diagnosis of vascular lesions (evidence level A).^4,13,14^ Doppler US is the most appropriate exam because of its cost-benefit profile, availability, and low invasivity, because it is radiation free, and because it has a good capacity for morphological and hemodynamic assessment of plaques and the degree of stenosis.^15,16^ Moreover, it has 99% sensitivity and 88% specificity for plaques exceeding 60%.^17^

Despite the method’s advantages, Doppler US screening is not recommended in asymptomatic patients in the adult population in general who have no significant risk factors^16^ (evidence level B and recommendation grade 1)^10^ (evidence level D).^18^ Although screening with Doppler US causes minimal direct harm, the method and other confirmatory techniques have imperfect sensitivity and specificity and may lead to false-positive diagnoses, primarily when conducted by inexperienced professionals, and can result in unnecessary interventions that may even cause displacement of plaques. Moreover, the examination involves costs, both for the patient and for the public or private health care provider, and if ordered inappropriately it does not offer benefits to the patient and does not reduce cerebral events in populations with low prevalence of carotid stenosis.^12,16^

In view of the complications of carotid stenosis secondary to atherosclerosis, its morbidity and mortality, and the high cost of treatment, the objective of this study was to evaluate whether medical specialists base their decisions on the Brazilian and international literature when ordering Doppler ultrasound screening of the carotids. The study also analyzed which medical specialties ordered the examination correctly most frequently.

## METHODOLOGY

Asymptomatic patients with risk factors for carotid stenosis were selected. Patients were selected retrospectively based on requests for carotid Doppler ultrasound at an angiology and vascular surgery clinic in the town of Cascavel (Paraná, Brazil), from January 2020 to January 2021. The sample size calculation assumed that 40% and 20% of Doppler US requests would be correct when made by vascular surgery specialists and other specialists, respectively. Using the formula described by Miot,^19^ considering α = 5% and β = 20%, the sample (n) for each group would be 78.4 (n = 79) participants, after rounding, which was not achieved, since we assessed a total of 152 patients, in which the sample (n) for each group would be 76.

The following baseline characteristics were recorded: age up to 80 years; male and female; SAH; current smoking or in remission (quit using tobacco up to 12 months previously); obesity; inactivity; DM; hyperlipidemia, characterized by total cholesterol > 200 mg/dL, LDL > 100 mg/dL or taking antihyperlipidemic agents; and congenital or acquired structural heart disease and asymptomatic or symptomatic coronary pathology whether treated or not, clinically and/or surgically.

The data collected were computed and analyzed with RStudio version 1.3.959. The results were tabulated and described in analysis and discussion of the results, demonstrating the reasons for requesting examinations and whether these were in accordance to the recommendations in the most recent guidelines.^4,10-12,16,20,21^ With relation to the main medical specialties that requested Doppler US and the accuracy of their requests, confidence intervals (CI) and p values were calculated using the chi-square test, adopting p < 0.05 for statistically relevant results. This article was approved by the Research Ethics Committee at the Centro Universitário da Fundação Assis Gurgacz, CAAE 38662320.6.0000.5219 and consolidated opinion number 4.650.756.

## RESULTS

A total of 200 requests for carotid Doppler ultrasound were identified during the study period. Excluding repeats and those missing information, 152 requests for patients aged up to 80 years were used for the study. Ninety-six of these patients were female (Table 1), and 47.37% were aged from 71 to 80 years (Table 1).

**Table 1:** Description of sex, age group, and characteristics such as smoking, associated comorbidities, clinical indications, and specialty of requesting physician (n = 152).

**Characteristic**	**Absolute frequency**	**Relative frequency**
Sex		
Female	96	63.16%
Male	56	36.84%
Age		
51 to 60 years	14	9.21%
61 to 70 years	66	43.42%
71 to 80 years	72	47.37%
Smoker or smoker in remission?		
Yes	14	9.21%
Denied	138	90.79%
Associated comorbidities		
Heart disease	12	7.89%
Coronary pathology	13	8.55%
Diabetes	37	24.34%
Hyperlipidemia	40	26.32%
Hypertension	62	40.79%
Inactivity	18	11.84%
Most prevalent indications		
Suspected atherosclerosis	23	15.13%
Other causes	14	9.21%
Vascular check-up	91	59.86%
Cervical and/or carotid murmurs	20	13.16%
Dizziness	4	2.63%
Specialty of professional who requested screening			
Cardiologist	7	4.61%	
Vascular surgeon	127	83.56%	
Endocrinologist	3	1.97%	
Geriatrician	12	7.89%	
Neurologist	3	1.97%	

Source: the authors (2021).

Hypertension was the most prevalent risk factor, affecting 62 individuals (Table 1). The next most common risk factor was hyperlipidemia.

The most common reason for requesting carotid Doppler ultrasound for carotid screening was vascular check-up, in 91 patients (Table 1). The medical specialties that most often requested carotid examinations were vascular surgery and geriatrics (Table 1).

With regard to the indications recorded for ordering carotid Doppler ultrasound, the request was judged to be appropriate if the patient had at least one risk factor for carotid plaques, excluding male sex. From the total sample, 97 requests were considered inappropriate (Table 2).

**Table 2:** Frequency of correct indications for ordering Doppler ultrasonography of the carotid arteries (n = 152).

**Characteristic**	**Absolute frequency**	**Relative frequency**
Correct indications?		
Yes	55	36.18%
No	97	63.82%

Source: the authors (2021).

Analysis of the rate of correct reasons for requesting carotid echography by specialty (Table 3) revealed that specialists in angiology and vascular surgery were correct more frequently than other types of health professionals (the odds ratio for correct indications for ordering the examination was 3.52 [CI 1.14-10.87], with p = 0.02).

**Table 3:** Comparison between vascular surgery and other medical specialties in terms of the accuracy of indications for ordering Doppler ultrasonography of the carotid arteries.

**Specialty**	**Correct indications**	**Incorrect indications**	**Odds ratio for correct indications (95%CI)**	***p*-value**
Vascular surgery	51 (40.16%A)	76 (59.84%B)	3.52 (1.14 - 10.87)	0.02*
Other specialties	4 (16%C)	21 (84%D)	Reference**	

Source: the authors (2021).

* Chi-square test.

** Other specialties were used as the reference category to calculate the odds ratios for correct indications.

A: correct indications as a percentage of total number of requests by vascular surgeons.

B: incorrect indications as a percentage of total number of requests by vascular surgeons.

C: correct indications as a percentage of total number of requests by other specialists.

D: incorrect indications as a percentage of total number of requests by other specialists.

## DISCUSSION

Asymptomatic stenosis of the carotid arteries is present in 2-18% of the population and the prevalence of stenosis > 70% is in the range of 0-3.1% in the general population. However, prevalence increases with age and if the patient has risk factors.^22^

The risk factors for moderate to severe carotid atherosclerosis are advanced age, SAH, obesity, current smoking or smoking in remission, hyperlipidemia, DM, male sex, inactivity, PAOD, coronary pathologies, and heart diseases - the first four of which have the strongest associations.^1,4,9,11^ The independent predictors of increased prevalence of asymptomatic stenosis are SAH, DM, hyperlipidemia, family history of stroke, PAOD, smoking, and left main coronary artery disease, the last three of which indicate stenosis ≥ 80%.^10^

Qureshi et al.^20^ selected 394 patients and used Doppler US screening to identify whether they had asymptomatic moderate carotid artery stenosis (> 50%). The patients screened were ≥ 60 years old and had at least one of the following risk factors: CAD, SAH, smoking, or history of AVC in first-degree relatives. The variables most associated with ≥ 60% carotid stenosis were age ≥ 65, current smoking, CAD, and hypercholesterolemia. Among the patients without risk factors, the prevalence of stenosis was 2%, increasing to 6% with one risk factor, to 14% with two risk factors, and to 16% with three risk factors.^23^

A meta-analysis analyzed 23,706 people from the general population with the objective of identifying the prevalence of carotid stenosis in asymptomatic patients, defining 50-69% stenosis as moderate and stenosis exceeding 70% as severe. They found the highest rates of stenosis among patients aged 70-79 years, in whom plaques > 50% were found in 6% of men and 3.61% of women, and plaques > 70% were found in 3.6 and 1% of men and women respectively.^24^ As also observed by Woo et al.,^11^ the age group with the highest frequency of carotid plaques and stenosis was 70 to 79 years.

Screening for carotid stenosis in asymptomatic patients is based on prevention of stroke, since this pathology can be the first sign of significant carotid atherosclerosis. The National Stroke Association, Canadian Stroke Consortium, and the United States Preventive Services Taskforce (USPSTF) agree that carotid screening reduces the risk of AVC when the prevalence of significant stenosis is ≥ 20%.^10^

According to the Brazilian Society of Angiology and Vascular Surgery (SBACV),^4^ Doppler US is recommended for initial screening in cases of asymptomatic patients with suspected carotid stenosis and/or risk factors for carotid stenosis. The primary candidate patients should be men with CAD, hyperlipidemia, and hypertension (evidence level B).^4^ The Atherosclerotic Disease of the Carotid Artery study suggests that screening should be conducted at ≥ 65 years when there is also CAD and/or history of smoking and/or hyperlipidemia,^25^ and the Guidelines for Screening of Extracranial Carotid Artery Disease state that screening should be performed in patients ≥ 65 years of age with at least three cardiovascular risk factors (evidence level A).^20^

Screening for asymptomatic carotid artery stenosis may be indicated for patients with more than one risk factor for vascular disease and carotid atherosclerotic disease, particularly if such individuals are inclined to consider carotid intervention if significant stenosis is discovered, in addition to the objective of controlling these factors and providing treatment to reduce morbidity and mortality (evidence level B and recommendation grade 2).^11,23^ According to AbuRahma et al.,^10^ patients in this group: are subjected to myocardial revascularization, are ≥ 55 years old, and have at least two risk factors for atherosclerosis, such as active smoking, diabetes, hypertension, or coronary pathologies and silent cerebral infarcts seen on imaging exams. Screening for carotid artery disease is strongly recommended in patients with symptomatic peripheral arterial disease, irrespective of age (evidence level A).^20^

However, routine screening is not recommended for all cases of asymptomatic carotid artery stenosis in the absence of significant risk factors (evidence level B and recommendation grade 1).^10^ A study by Anne et al. reported that just 5.5% of asymptomatic individuals free from prior pathologies will suffer a stroke at some point in life because of asymptomatic carotid stenosis.^26^

In addition to increasing the risk of stroke, carotid stenosis increases risk of cardiac mortality.^12^ Screening of patients with asymptomatic but significant acute coronary syndrome (ACS) with carotid Doppler ultrasound is important to reclassify these patients’ late cardiac risk and to analyze whether they need prospective follow-up, medication, or surgical treatment.^11^ The First Brazilian Cardiovascular Prevention Guideline and the Fifth Brazilian Dyslipidemia and Atherosclerosis Prevention Guideline, cited by Freire et al.,^17^ recommend that subclinical carotid atherosclerosis detected by imaging methods should be interpreted as a criterion for identification of patients at high risk of coronary events. The American College of Cardiology/American Heart Association guidelines recommend that screening should be considered for patients who will undergo myocardial revascularization surgery and are aged ≥ 65 years, or have left main coronary artery stenosis, history of smoking, TIA, stroke, carotid murmur, or PAOD.^10^

The main reasons for not recommending carotid stenosis screening of the general population include associated harms, since patients may undergo unnecessary interventions that trigger a procedure-related stroke.^18^ As such, a positive ultrasonographic screening result should not necessarily be interpreted as an indication for surgery.^12^

Although carotid US for screening and diagnosis is a widely available method, indiscriminately ordering it imposes considerable expense, both on the patient, and on the public or private health care provider, taking into account the moderate to high cost of the examination, the economic situation in Brazil, and the fact that the majority of these patients are elderly, with other pathologies that also increase their spending on health care.^12,16^

Other factors that support not screening the general population include the failure to prove a reduction in cerebral events, since stenosis is not the only etiology of stroke, and the large number of false-positive tests, particularly when performed by inexperienced professionals and in populations with significant prevalence ≤ 5%.^12^

Females were more prevalent in the population analyzed in the present study, as was the case in a study by Freitas et al.^1^ Patients ≥ 60 years of age accounted for more than 90% of the asymptomatic patients for whom US of the carotid arteries was ordered. This was expected, since the presence of risk factors for significant asymptomatic stenosis increases with the age of the population. In agreement with Freitas et al.,^1^ hypertension was the most prevalent risk factor in this study population, followed by hypercholesterolemia and DM.

The most common reason given for ordering US of the carotid arteries of asymptomatic patients at the clinic where this study was conducted was for vascular check-up because the patients had other vascular changes; in 40.14% of the patients the reason for ordering a check-up was suspicion of atherosclerotic carotid disease, concomitant with PAOD, advanced coronary disease, or aneurysms, or simply because the patient had several risk factors for atherosclerosis. As stated by AbuRahma et al.,^10^ cervical murmurs are a criterion for carotid screening in asymptomatic patients. Other reasons put on the requests included dizziness, syncope, general check-up, and non-atherosclerotic concomitant vascular diseases. It should be mentioned that the American syncope guideline concluded that US should not be ordered for all asymptomatic patients with syncope (recommendation grade 3 - no benefit).^21^

On the basis that the indications for ordering US screening for carotid artery stenosis in asymptomatic patients are considered correct if the patient has one or more risk factors or criteria for requesting the examination in the population, the majority of requests assessed in the present study were not supported by the literature. It was found that just 16% of the indications from specialties other than vascular surgery were correct, compared to 40.16% of those from vascular surgeons (odds ratio for correct indications of 3.52 [CI 1.14-10.87], p = 0.02). Analyzing requests for US screening for carotid artery stenosis submitted by specialists in vascular surgery and the other specialists assessed in the study, the overall percentage of correct indications was 36% (95%CI 29-42%).

The information reported in this study demonstrates that medical specialists who care for patients with carotid stenosis should adequately recognize the indications for this examination in order to avoid over-prescription, false diagnoses, and iatrogeny. As such, it is important for the medical community that all specialists — and not only vascular surgeons — improve their knowledge about carotid diseases.

This study has some limitations that should be considered. The study was carried out at a single angiology and vascular surgery clinic and so the number of Doppler ultrasound examinations requested by vascular surgeons was greater than the number ordered by the other medical specialties, which may have contributed to their higher rate of correct indications. The estimated sample size (n) was not attained for either group (n = 79), which is another limitation. The number of participants whose examinations were ordered by other specialties was n = 25, which is a large disparity compared with the 127 requests made by vascular surgeons, which means that an adequate comparison between these groups cannot be made. Another limitation is that it is not possible to infer whether there might be some factor that could explain the number of inappropriate carotid Doppler ultrasound requests. In order to correct these biases, further studies could be conducted at radiology clinics, where there would be a more balanced influx of requests for Doppler ultrasound from the different medical specialties and the chance to assess a larger sample.

## CONCLUSIONS

It can be concluded that the specialists ordered an excessive number of carotid US examinations for screening. Vascular surgeons were more often correct in their indications for carotid US to screen for the presence of stenosis (odds ratio for correct indications of 3.52 [CI 1.14-0.87], with p = 0.02), while specialists other than vascular surgeons correctly ordered 16% of their carotid artery US requests. Notwithstanding, 59.8% of the requests by vascular surgeons were not in line with the literature and, therefore, in common with the non-vascular surgeon specialists, all specialists should base their conduct on the literature, in order to order carotid US correctly.

Although it was not possible to infer whether the incorrect requests were because of failure to base them on the literature, it is essential to emphasize the importance of evidence-based medicine and recourse to the literature when ordering carotid screening to limit its use to those who will actually benefit.
