# Supplementary material for: Indications for carotid Doppler ultrasound in asymptomatic patients - are we ordering it correctly?
Source: J Vasc Bras. 2023 Aug 7;22:e20220084. doi: 10.1590/1677-5449.202200841 (PMC10421587; doi:10.1590/1677-5449.202200841)
Supplement: Translation

# Translation: Indicações para ecodoppler de carótidas em pacientes assintomáticos - estamos solicitando corretamente?

**DOI:** 10.1590/1677-5449.202200842

**Authors:** Alcides Alves da Silva, André Brusamolin Moro, Jeferson Freitas Toregeani

**Affiliations:** 1 Centro Universitário da Fundação Assis Gurgacz - FAG, Cascavel, PR, Brasil.; 2 Universidade Estadual do Oeste do Paraná - UNIOESTE, Cascavel, PR, Brasil.

**Keywords:** aterosclerose carotídea assintomática, *screening* de estenose carotídea assintomática, ultrassonografia de artérias carótidas

## Abstract

**Contexto:**

Artérias carótidas são locais de frequentes patologias, sendo a mais comum a aterosclerose, podendo resultar na formação de placas com estenose. Para avaliarmos as carótidas em pacientes assintomáticos, o método de ultrassonografia Doppler ou ecodoppler é, atualmente, o exame de eleição, indicado para a triagem e o diagnóstico de lesões vasculares. As diretrizes atuais recomendam o rastreamento em pacientes que apresentem fatores de risco para estenose carotídea e que estejam aptos e dispostos ao tratamento medicamentoso/intervenção carotídea. A triagem em pacientes assintomáticos na população adulta em geral e sem fatores de risco significativos não é recomendada.

**Objetivos:**

Avaliar se os especialistas médicos se baseiam na literatura para solicitar o ecodoppler como *screening*.

**Métodos:**

Realizou-se seleção retrospectiva dos pacientes a partir da solicitação de ecodoppler. Os dados coletados foram computados e analisados com o programa RStudio versão 1.3.959.

**Resultados:**

Avaliou-se a solicitação como adequada, desde que os pacientes apresentassem pelo menos um fator de risco para placas carotídeas. Entre 152 pacientes, 55 preenchiam critérios para *screening* de carótidas, a principal indicação na população estudada foi o *check-up* vascular. Hipertensão arterial foi o fator de risco mais prevalente. Os especialistas em cirurgia vascular obtiveram êxito maior ao solicitar o exame de maneira adequada [razão de chances de indicações corretas de 3,52 (IC 1,14-10,87), com p = 0,02]. A assertividade nas indicações de ecodoppler foi de 36% (IC95% 29-42%).

**Conclusões:**

Conclui-se que houve excesso de solicitação de ultrassonografia de carótidas como *screening.* Os cirurgiões vasculares solicitaram o exame de maneira mais assertiva.

## INTRODUÇÃO

As artérias carótidas são locais de frequentes alterações patológicas desde o nascimento até a velhice, sendo a mais comum a estenose causada por aterosclerose^1^ . Tal patologia tem predileção por determinadas artérias, afetando as carótidas, principalmente ao nível da bifurcação e porção inicial da carótida interna^2^ . A aterosclerose pode resultar na formação de placas com variadas morfologias e características, que podem estenosar e obstruir a carótida ou resultar em embolização para o encéfalo^3-5^ .

Os principais fatores de risco para doença carotídea extracraniana incluem: idade avançada, hipertensão arterial sistêmica (HAS), tabagismo, hipercolesterolemia, diabetes melito (DM), sexo masculino, obesidade, doença arterial obstrutiva periférica (DAOP), doenças arteriais coronarianas (DAC) e cardíacas^6-8^ . A aterogênese, seguida da formação de placas ateromatosas, está relacionada a eventos cerebrovasculares^9^ .

Como consequência do grau de estenose e da embolização a partir da placa ateromatosa, o fluxo sanguíneo cerebral pode ser comprometido, limitando a oferta de oxigênio e glicose e podendo acarretar em acidente vascular encefálico (AVE), acidente isquêmico transitório (AIT), amaurose fugaz e outras condições neurológicas^6^ . A triagem de estenose carotídea em pacientes assintomáticos baseia-se na prevenção de AVE, podendo essa ser a primeira evidência de aterosclerose carotídea significativa^10^ .

Devido à gravidade da estenose carotídea de longa data não diagnosticada, métodos de *screening* são essenciais à prática clínica. De acordo com o *guideline* da European Society for Vascular Surgery (ESVS), o *screening* de estenose carotídea assintomática é indicado para pacientes com fatores de risco para aterosclerose, a fim de otimizar o controle desses fatores, reduzir a morbimortalidade e evitar procedimentos invasivos^11^ . A ESVS, em 2011, recomendou que o rastreamento seja feito em grupos de pacientes que apresentem fatores de risco para estenose arterial carotídea, porém, tais pacientes devem estar aptos e dispostos ao tratamento medicamentoso e/ou à intervenção carotídea se estenose significativa for diagnosticada^12^ .

Para a avaliação das carótidas em pacientes assintomáticos, o método de ultrassonografia (US) Doppler (ou ecodoppler) de carótidas é, atualmente, o exame de eleição, sendo amplamente disponível e indicado para a triagem e diagnóstico de lesões vasculares (Nível de evidencia A)^4,13,14^ . A US Doppler é o exame mais indicado pelo melhor custo-benefício, disponibilidade, por ser menos invasivo, não apresentar radiação e por ter boa capacidade de avaliação morfológica e hemodinâmica das placas e o grau de estenose^15,16^ . Soma-se a isso a sensibilidade de 99% e a especificidade de 88% para placas > 60%^17^ .

Apesar das vantagens desse método, a US Doppler como triagem em pacientes assintomáticos na população adulta em geral e sem fatores de risco significativo não é recomendada^16^ (Nível de evidência B e Grau de recomendação 1)^10^ (Nível de evidencia D)^18^ . Embora a triagem com US Doppler cause mínimos danos diretos, esse método e outros confirmatórios apresentam sensibilidade e especificidade imperfeitas, podendo levar a diagnósticos falso-positivos, principalmente quando realizados por profissionais inexperientes, e podendo acarretar em intervenções desnecessárias que podem desencadear o deslocamento de placas. Além disso, é um exame custoso tanto para o paciente quanto para a saúde pública e suplementar que, quando inadequadamente indicado, não traz benefícios ao paciente e não reduz eventos cerebrais nas populações com baixa prevalência de estenose carotídea^12,16^ .

Tendo em vista as complicações da estenose carotídea secundária a aterosclerose, a morbimortalidade e o alto custo do seu tratamento, este estudo teve o objetivo de avaliar se os especialistas médicos se baseiam na literatura nacional e internacional para solicitar o ecodoppler de carótidas como *screening*. Além disso, analisaram-se quais eram as especialidades médicas mais assertivas na solicitação do exame.

## METODOLOGIA

Foram selecionados pacientes assintomáticos com fatores de risco para estenose carotídea. A seleção dos pacientes deu-se de forma retrospectiva, a partir da solicitação de ecodoppler de carótidas em uma clínica de angiologia e cirurgia vascular na cidade de Cascavel (Paraná, Brasil), de janeiro de 2020 a janeiro de 2021. Para o cálculo do tamanho da amostra, assumiu-se que 40% e 20% das indicações realizadas pela cirurgia vascular e de outras especialidades eram corretas, respectivamente. Utilizando a fórmula descrita no trabalho de Miot^19^ , considerando-se α = 5% e β = 20%, o n amostral para cada grupo seria de 78,4 (n = 79) participantes após arredondamento, o qual não foi atingido, pois avaliamos as solicitações de 152 pacientes, em que o n amostral de cada grupo seria de 76.

As seguintes características basais foram registradas: idade até 80 anos; sexo masculino e feminino; HAS; tabagismo atual ou em remissão (cessação do uso de tabaco em até 12 meses); obesidade; sedentarismo; DM; hiperlipidemia caracterizada por colesterol total > 200 mg/dL, LDL > 100 mg/dL ou em uso de medicação hipolipemiante; cardiopatia estrutural congênita ou adquirida e coronariopatia assintomática ou sintomática tratada ou não clinicamente e/ou cirurgicamente.

Os dados coletados foram computados e analisados com o programa RStudio versão 1.3.959. Os resultados foram compilados em tabelas, descritas em análise e discussão dos resultados, demonstrando o motivo da solicitação e se esta estava de acordo com o que consta em diretrizes (*guidelines*)^4,10-12,16,20,21^ mais recentes. Com relação às principais especialidades médicas que solicitaram o exame e a acurácia na solicitação, foi calculado o intervalo de confiança (IC) e o valor de p por meio do teste qui-quadrado, sendo considerado p < 0,05 para resultados estatisticamente relevantes. O presente artigo foi aprovado pelo Comitê de Ética em Pesquisa do Centro Universitário da Fundação Assis Gurgacz, sob CAAE 38662320.6.0000.5219 e do Parecer Consubstanciado, sob número 4.650.756.

## RESULTADOS

Durante o período do estudo, 200 solicitações de ecodoppler de carótidas foram encontradas. Excluindo-se as repetidas e com informações insuficientes, 152 solicitações de pacientes com idade de até 80 anos foram utilizadas. Do total de pacientes, 96 eram do sexo feminino (Tabela 1), e 47,37% estavam na faixa etária entre 71-80 anos de idade (Tabela 1).

**Tabela 1:** Descrição de sexo, faixa etária, características como tabagismo, comorbidades associadas, indicação clínica e especialidade do médico solicitante (n = 152).

**Característica**	**Frequência absoluta**	**Frequência relativa**
Sexo		
Feminino	96	63,16%
Masculino	56	36,84%
Idade		
51 a 60 anos	14	9,21%
61 a 70 anos	66	43,42%
71 a 80 anos	72	47,37%
Tabagista ou tabagismo em remissão?		
Sim	14	9,21%
Nega	138	90,79%
Comorbidades associadas		
Cardiopatia	12	7,89%
Coronariopatia	13	8,55%
Diabetes	37	24,34%
Hiperlipidemia	40	26,32%
Hipertensão	62	40,79%
Sedentarismo	18	11,84%
Indicações mais prevalentes		
Suspeita de aterosclerose	23	15,13%
Outras causas	14	9,21%
*Check-up* vascular	91	59,86%
Sopros cervicais e/ou carotídeos	20	13,16%
Tontura	4	2,63%
Especialidade do profissional que solicitou			
Cardiologista	7	4,61%	
Cirurgião vascular	127	83,56%	
Endocrinologista	3	1,97%	
Geriatra	12	7,89%	
Neurologista	3	1,97%	

Fonte: Autores (2021).

A hipertensão foi o fator de risco mais prevalente, acometendo 62 indivíduos (Tabela 1). Entre os demais fatores de risco, destaca-se a hiperlipidemia.

Em relação às solicitações de ecodoppler de carótidas, a principal indicação para a realização do *screening* carotídeo foi o *check-up* vascular em 91 pacientes (Tabela 1). As especialidades médicas que mais solicitaram o exame de carótidas foram a cirurgia vascular, seguida pela geriatria (Tabela 1).

Quanto às indicações do ecodoppler de carótidas, avaliou-se a solicitação como adequada, desde que os pacientes apresentassem pelo menos um fator de risco para placas carotídeas, exceto sexo masculino. Do total, 97 das indicações foram consideradas inadequadas (Tabela 2).

**Tabela 2:** Assertividade na indicação do exame de ultrassonografia de artérias carótidas com Doppler (n = 152).

**Característica**	**Frequência absoluta**	**Frequência relativa**
Indicação correta?		
Sim	55	36,18%
Não	97	63,82%

Fonte: Autores (2021).

Sobre a assertividade das especialidades médicas na solicitação da ecografia de carótidas (Tabela 3), observou-se que os especialistas em angiologia e cirurgia vascular obtiveram êxito maior que os demais profissionais para solicitar o exame adequadamente (razão de chances de indicações corretas de 3,52 (IC 1,14-10,87), com p = 0,02).

**Tabela 3:** Comparação entre cirurgia vascular e outras especialidades médicas na acurácia da indicação do exame de ultrassonografia com Doppler de artérias carótidas.

**Especialidade**	**Indicações corretas**	**Indicações incorretas**	**Razão de chances de indicações corretas (IC95%)**	**p valor**
Cirurgia vascular	51 (40,16%A)	76 (59,84%B)	3,52 (1,14 - 10,87)	0,02*
Outras especialidades	4 (16%C)	21 (84%D)	Referência**	

Fonte: Autores (2021).

* Teste de qui-quadrado.

** As outras especialidades foram utilizadas como referência para o cálculo da razão de chances de indicações corretas.

A: porcentagem de indicações corretas referentes ao total de indicações de cirurgiões vasculares.

B: porcentagem de indicações incorretas referentes ao total de indicações de cirurgiões vasculares.

C: porcentagem de indicações corretas referentes ao total de indicações de outras especialidades.

D: porcentagem de indicações incorretas referentes ao total de indicações de outras especialidades.

## DISCUSSÃO

A estenose de artérias carótidas assintomáticas está presente em 2-18% da população e, se considerada apenas a estenose > 70%, a prevalência varia em torno de 0-3,1% na população geral. Porém, essa prevalência aumenta com a idade e se o paciente apresentar fatores de risco^22^ .

Os fatores de risco para aterosclerose carotídea moderada a grave são idade avançada, HAS, obesidade, tabagismo atual ou em remissão, hiperlipidemia, DM, sexo masculino, sedentarismo, DAOP, coronariopatias e cardiopatias, sendo os quatro primeiros os mais associados^1,4,9,11^ . Entre esses, os preditores independentes para prevalência aumentada de estenose assintomática são HAS, DM, hiperlipidemia, histórico familiar de AVE, DAOP, tabagismo e doença de tronco de coronária esquerda, sendo que os três últimos indicam estenose ≥ 80%^10^ .

Qureshi et al.^20^ selecionaram 394 pacientes a fim de identificar se apresentavam estenose de artéria carótida assintomática moderada (> 50%) via triagem com US Doppler. Os pacientes examinados tinham ≥ 60 anos de idade e pelo menos um dos seguintes fatores de risco: DAC, HAS, tabagismo e história de AVC em parentes de primeiro grau. As principais variáveis associadas com ≥ 60% de estenose carotídea foram idade ≥ 65, tabagismo atual, DAC e hipercolesterolemia. Nos pacientes sem fatores de risco, a prevalência de estenose foi de 2%; com um fator de risco, de 6%; se dois fatores de risco, de 14%; e, com três, de 16%^23^ .

Uma metanálise avaliou 23.706 pessoas da população geral com o objetivo de identificar a prevalência de estenose carotídea em pacientes assintomáticos, considerando estenose moderada entre 50-69% e grave quando maior que 70%. Encontraram-se maiores taxas de estenose entre os pacientes com 70-79 anos de idade, em que placas > 50% foram encontradas em 6% dos homens e em 3,61% das mulheres, e as > 70% foram encontradas em 3,6 e 1%, respectivamente^24^ . Assim como observado por Woo et al.^11^ , a faixa etária com maior frequência de placa e estenose carotídea foi o grupo de 70 a 79 anos.

A triagem de estenose carotídea em pacientes assintomáticos baseia-se na prevenção de AVE, em que tal patologia pode ser a primeira evidência de aterosclerose carotídea significativa. O National Stroke Association, Canadian Stroke Consortium e a Força-Tarefa de Serviços Preventivos dos Estados Unidos (USPSTF) concordam que o *screening* de carótidas reduz o risco de AVC quando a prevalência de estenose significativa for ≥ 20%^10^ .

Segundo a Sociedade Brasileira de Angiologia e Cirurgia Vascular (SBACV)^4^ , em caso de paciente assintomático, mas com suspeita de estenose carotídea e/ou fatores de risco para tal, a US Doppler é recomendado como triagem inicial. Os principais pacientes candidatos devem ser homens com DAC, hiperlipidêmicos e hipertensos (Nível de evidência B)^4^ . O estudo *Atherosclerotic Disease of the Carotid Artery* sugere que a triagem seja feita em ≥ 65 anos de idade com associação a DAC e/ou história de tabagismo e/ou hiperlipidemia^25^ , e o *Guidelines for Screening of Extracranial Carotid Artery Disease* relata que o *screening* deve ser feito em pacientes ≥ 65 anos com pelo menos três fatores de risco cardiovascular (Nível de evidência A)^20^ .

A triagem para estenose de artéria carotídea assintomática seletiva pode ser indicada para pacientes com mais de um fator de risco vascular e para doença aterosclerótica carotídea, particularmente, se os indivíduos estiverem dispostos a considerar a intervenção carotídea em caso de presença de estenose significativa, além de objetivar o controle de tais fatores e a terapêutica para reduzir a morbimortalidade (Nível de evidência B e Grau de recomendação 2)^11,23^ . De acordo com AbuRahma et al.^10^ , os pacientes que se enquadram nesse grupo: são submetidos a revascularização do miocárdio, têm ≥ 55 anos e pelo menos dois ou mais fatores de risco para aterosclerose, como tabagismo ativo, diabéticos, hipertensos ou coronariopatas e infartos cerebrais silenciosos vistos em exames de imagem. A pacientes com doença arterial periférica sintomática, independentemente da idade, recomenda-se fortemente a triagem para doença arterial carotídea (Nível de evidencia A)^20^ .

Entretanto, a triagem de rotina para todos os casos de estenose de artérias carótidas assintomáticas e sem fatores de risco significativo não é recomendada (Nível de evidência B e Grau de recomendação 1)^10^ . O estudo de Anne et al. relatou que apenas 5,5% dos indivíduos assintomáticos e sem patologias prévias evoluirão com AVE ao longo da vida devido a estenose carotídea assintomática^26^ .

A estenose carotídea, além de aumentar o risco de AVE, também eleva o risco de mortalidade cardíaca^12^ . A triagem com ecodoppler de carótidas em pacientes com síndrome coronariana aguda (SCA) assintomática, mas significativa, é importante para reclassificar o risco cardíaco de tais pacientes e analisar se eles necessitarão de acompanhamento longitudinal, medicação ou tratamento cirúrgico^11^ . A I Diretriz Brasileira de Prevenção Cardiovascular e a V Diretriz Brasileira de Dislipidemias e Prevenção da Aterosclerose, citadas por Freire et al.^17^ , recomendam que a presença de aterosclerose carotídea subclínica, detectada por metodologia de imagem, seja interpretada como critério de identificação de pacientes em alto risco de eventos coronarianos. As diretrizes do American College of Cardiology/American Heart Association recomendam que a triagem seja considerada para pacientes que serão submetidos a cirurgia de revascularização do miocárdio e que tenham ≥ 65 anos, presença de estenose em tronco de coronária esquerda, histórico de tabagismo, AIT, AVE, sopro carotídeo ou DAOP^10^ .

Entre os principais motivos para não se recomendar a triagem de estenose carotídea na população geral, incluem-se os danos associados, pois os indivíduos podem sofrer intervenções desnecessárias e desencadear um AVC associado ao procedimento^18^ . Desse modo, uma triagem ultrassonográfica positiva não deveria, necessariamente, ser interpretada como indicação cirúrgica^12^ .

Apesar de a US de carótidas como método de triagem e diagnóstico ser bastante disponível, sua solicitação indiscriminada acarreta alto gasto, seja por parte do paciente, seja por parte da saúde pública ou suplementar, tendo em vista o valor moderado a alto do exame, o cenário econômico em nosso país e por a maioria dos pacientes ser de idosos com outras patologias que elevam ainda mais os gastos com a saúde^12,16^ .

Outros fatores que corroboram a não realização da triagem na população geral são a falta de comprovação da redução de eventos cerebrais, já que a estenose não é a única etiologia de AVE, e o grande número de testes falso-positivos, principalmente quando feito por profissionais inexperientes e em populações com prevalência significativa ≤ 5%^12^ .

Na população analisada pelo presente estudo, o sexo feminino foi o mais representativo, assim como no estudo de Freitas et al.^1^ . Pacientes ≥ 60 anos respondem por mais de 90% dos indivíduos assintomáticos que tiveram solicitação de US de artérias carótidas. Isso era esperado, pois a presença de fatores de risco para estenose assintomática significativa na população aumenta com a idade. Em concordância com Freitas et al.^1^ , a hipertensão foi o fator de risco mais prevalente na população estudada, seguida por hipercolesterolemia e DM.

Entre os motivos para a solicitação de US de artérias carótidas em pacientes assintomáticos na clínica em que foi realizado este estudo, a principal justificativa foi o *check-up* vascular, devido aos pacientes apresentarem outras alterações vasculares; em 40,14% dos pacientes, a indicação foi devida à suspeita de doença carotídea aterosclerótica, em concomitância com DAOP, doença coronariana avançada ou presença de aneurismas ou apenas por apresentar vários fatores de risco para aterosclerose. Sopros cervicais, como relatado por AbuRahma et al., são um critério para a triagem de carótidas em assintomáticos^10^ . Outras justificativas presentes nas solicitações avaliadas foram tontura, síncope, *check-up* geral e doenças vasculares não ateroscleróticas concomitantes. Vale ressaltar que o *guideline* americano de síncope concluiu que a US não deve ser solicitada para todos os pacientes assintomáticos que apresentarem síncope (Grau de recomendação 3 - sem benefício)^21^ .

Considerando como corretas as solicitações de US para a triagem de estenose em artérias carótidas de pacientes assintomáticos, aquelas em que o indivíduo apresentava um ou mais fatores de risco ou critérios para solicitação do exame na população, a maioria das solicitações não se embasou na literatura, de acordo com o encontrado no atual estudo. Verificou-se que somente 16% das indicações dos especialistas não cirurgiões vasculares foram corretas, comparando-se a 40,16% dos cirurgiões vasculares (Razão de chances de indicações corretas de 3,52 (IC 1,14-10,87), p = 0,02). Analisando as solicitações de US para a triagem de estenose carotídea tanto pelos especialistas em cirurgia vascular quanto por outros especialistas avaliados no estudo, constata-se que o percentual de indicações corretas foi de 36% (IC95% 29-42%).

As informações deste estudo demonstram que as especialidades médicas que acompanham pacientes com estenose carotídea necessitam reconhecer adequadamente as indicações do exame a fim de evitar *overprescription*, falsos diagnósticos e iatrogenias. Desse modo, é de importância para a comunidade médica que todas as especialidades — e não apenas os cirurgiões vasculares — aprofundem o conhecimento sobre doenças carotídeas.

Este estudo teve algumas limitações que devem ser consideradas. O estudo foi realizado em uma clínica de angiologia e cirurgia vascular e, desse modo, o número de solicitações de ecodoppler realizadas por cirurgiões vasculares foi maior quando comparado às outras especialidades médicas, o que pode ter contribuído para a maior assertividade desses nas indicações do exame. O n amostral de cada grupo (n = 79) não foi atingido, sendo mais uma limitação deste estudo. A quantidade de participantes com indicações de outras especialidades foi de n = 25, havendo uma grande disparidade em comparação com as 127 solicitações feitas por cirurgiões vasculares, o que não permite fazer uma adequada comparação entre os grupos. Outra limitação é a impossibilidade de inferir se houve algum fator que possa explicar a quantidade de solicitações inadequadas de ecodoppler de carótidas. A fim de corrigir tais vieses, novos estudos podem ser realizados em clínicas de radiologia, onde há um fluxo mais equivalente de solicitações de ecodoppler das diversas especialidades médicas, assim como a possibilidade de incorporar uma amostragem maior.

## CONCLUSÃO

Conclui-se que existe excesso de solicitação de US de carótidas como *screening* por parte dos especialistas. Os cirurgiões vasculares obtiveram maior assertividade na indicação da US de carótidas como triagem para avaliar a presença de estenose (Razão de chances de indicações corretas de 3,52 (IC 1,14-0,87), com p = 0,02), e especialistas não cirurgiões vasculares foram assertivos em 16% de suas indicações de US de artérias carótidas. Entretanto, 59,8% das solicitações feitas por cirurgiões vasculares estão em desacordo com a literatura, portanto, assim como os especialistas não cirurgiões vasculares, todos os especialistas devem se basear na literatura, a fim de solicitar corretamente a US de carótidas.

Mesmo com a impossibilidade de inferir que as solicitações incorretas ocorreram devido à falta de embasamento na literatura, ressalta-se a importância da medicina baseada em evidências e do embasamento na literatura para solicitar o *screening* de carótidas para aqueles que realmente se beneficiarão dessa triagem.
